# Supplementary material for: Screening for Metallo-Beta-Lactamases Using Non-Carbapenem Agents: Effective Detection of MBL-Producing Enterobacterales and Differentiation of Carbapenem-Resistant Enterobacterales
Source: Antibiotics (Basel). 2023 Jul 3;12(7):1146. doi: 10.3390/antibiotics12071146 (PMC10376669; doi:10.3390/antibiotics12071146)
Supplement: Supplementary file 1 [file antibiotics-12-01146-s001.zip › Table S1.pdf]

Supplementary Table S1. non-duplicate sequential 3776 isolates

Bacterial isolarates	n=3776
Escherichia coli	1263
Klebsiella pneumoniae	653
Enterobacter cloacae	435
Klebsiella oxytoca	229
Serratia marcescens	221
Enterobacter aerogenes	217
Proteus mirabilis	130
Morganella morganii	114
Citrobacter freundii	101
Klebsiella ornithinolytica	96
Citrobacter koseri	69
Proteus vulgaris	59
Citrobacter braakii	45
Citrobacter werkmanii	14
Serratia liquefaciens	14
Klebsiella sp.	13
Providencia rettgeri	11
Edwardsiella tarda	9
Citrobacter sp.	8
Pantoea sp.	8
Proteus penneri	8
Citrobacter youngae	7
Klebsiella planticola	6
Citrobacter amalonaticus	5
Enterobacter sp.	5
Enterobacter gergoviae	4
Pantoea agglomerans	4
Leclercia adecarboxylata	3
Pasteurella canis	3
Providencia stuartii	3
Enterobacter sakazakii	2
Kluyvera cryocrescens	2
Proteus sp.	2
Yersinia enterocolitica	2

Capnocytophaga ochracea	1
Enterobacter amnigenus	1
Escherichia hermannii	1
Hafnia alvei	1
Klebsiella terrigena	1
Kluyvera intermedia	1
Kluyvera sp.	1
Plesiomonas shigelloides	1
Rahnella aquatilis	1
Salmonella sp.	1
Serratia odorifera	1
Total	3776
