# Supplementary material for: Screening for Metallo-Beta-Lactamases Using Non-Carbapenem Agents: Effective Detection of MBL-Producing Enterobacterales and Differentiation of Carbapenem-Resistant Enterobacterales
Source: Antibiotics (Basel). 2023 Jul 3;12(7):1146. doi: 10.3390/antibiotics12071146 (PMC10376669; doi:10.3390/antibiotics12071146)
Supplement: Supplementary file 1 [file antibiotics-12-01146-s001.zip › Table S2.pdf]

Supplementary Table S2. Classification of CRE strains based on drug susceptibility to ceftazidime and cefoperazone/sulbactam.

	Metallo beta lactamase	
	Positive(n=14)	Negative(n=146)
Resistant to both CAZ and CFP/SBT	14	18
Susceptible to either CAZ or CFP/SBT	0	128

Abbreviations: CAZ, ceftazidime; CFP/SBT, cefoperazone and sulbactam

There were 160 strains of CRE, which were classified into a four-tiered distribution based on drug susceptibility to ceftazidime and cefoperazone/sulbactam. All MBL-producing CRE strains showed resistance to ceftazidime and cefoperazone/sulbactam. For detailed information on MBL-producing CRE, please refer to Table 2. There were 18 non-MBL-producing CRE strains were resistant to both ceftazidime and cefoperazon/sulbactam and 128 non-MBL-producing CRE strains susceptible to either ceftazidime or cefoperazone/sulbactam. We classified the CRE isolates based on MBL-positive or -negative status and their resistance/susceptibility to two drugs in the four-cell table. Sensitivity and specificity were analysed based on this table. The isolates susceptible to both drugs did not include any MBL, and the statistical specificity and sensitivity were 87.7% and 100% respectively with statistical significance using Fisher's exact test ($P < 0.01$).
